# Risk factors and performance of coronary aneurysm risk scores in children with Kawasaki disease

**DOI:** 10.3389/fped.2026.1915826

**Published:** 2026-07-23

**Authors:** Cem Çanakçi, Halil Özdemir, Gül Arga, Hatice Kübra Konca, Esra Çakmak Taşkin, Tayfun Uçar, Ergin Çiftçi, Hasan Ercan Tutar

**Affiliations:** 1 Department of Pediatric Hematology and Oncology, Ankara University Faculty of Medicine, Ankara, Türkiye; 2 Department of Pediatric Infectious Diseases, Ankara University Faculty of Medicine, Ankara, Türkiye; 3 Department of Pediatric Infectious Diseases, Ankara Bilkent City Hospital, Ankara, Türkiye; 4 Department of Pediatric Infectious Diseases, Kocaeli City Hospital, Kocaeli, Türkiye; 5 Department of Pediatric Cardiology, Ankara University Faculty of Medicine, Ankara, Türkiye

**Keywords:** coronary artery abnormalities, coronary artery aneurysm, kawasaki disease, pediatrics, risk assessment, risk factors

## Abstract

**Purpose:**

Kawasaki disease (KD) is the leading cause of acquired heart disease in children, and coronary artery aneurysm (CAA) remains its most serious complication. Several clinical risk scoring systems have been developed to predict coronary involvement, mainly in East Asian populations, but their performance varies across different settings. This study aimed to identify clinical predictors of CAA and to evaluate the performance of commonly used risk scoring systems in a Turkish pediatric cohort.

**Methods:**

This retrospective single-center study included children diagnosed with KD between 1994 and 2019 in tertiary reference center of Türkiye. Demographic, clinical, and laboratory data were analyzed. CAA was defined as a coronary artery z-score ≥2.5. Established risk scoring systems were evaluated for their ability to identify patients with CAA.

**Results:**

Eighty-one patients were included, and CAA developed in 18 (22.2%). Younger age at diagnosis, prolonged fever duration, and delayed intravenous immunoglobulin (IVIG) treatment were more frequently observed among patients with CAA. Commonly used risk scores showed low sensitivity and limited overall performance in identifying patients with coronary involvement in this cohort.

**Conclusions:**

In this Turkish pediatric cohort, established Kawasaki disease risk scoring systems demonstrated variable diagnostic performance for predicting coronary artery aneurysm, with no single scoring system providing consistently balanced sensitivity and specificity. Age at diagnosis, fever duration, and timing of IVIG treatment may provide useful clinical indicators during patient assessment.

## Highlights

- Coronary artery aneurysm is the most important complication of Kawasaki disease.
- Several clinical risk scoring systems have been developed primarily to predict IVIG resistance, mainly in East Asian populations.
- The performance of these scoring systems in non-Asian populations remains inconsistent.
- In this Turkish pediatric cohort, commonly used Kawasaki disease risk scoring systems demonstrated variable and overall limited performance for prediction of coronary artery aneurysm.
- Younger age, prolonged fever duration, and delayed IVIG treatment were associated with coronary artery aneurysm development.
- These findings support the need for population-specific approaches when evaluating coronary artery risk in Kawasaki disease.

## 1 Introduction

Kawasaki disease (KD) is an acute, self-limited vasculitis of childhood and remains the leading cause of acquired heart disease in developed countries. Despite timely treatment, coronary artery involvement continues to be the most serious complication, potentially leading to coronary artery aneurysm (CAA), myocardial ischemia, and long-term cardiovascular morbidity (1). Early identification of patients at increased risk for coronary complications is therefore a major clinical priority.

Several clinical and laboratory-based scoring systems have been developed in patients with KD, particularly in East Asian populations. Most of these scoring systems, including the Kobayashi, Egami, and Sano scores, were originally designed to predict resistance to intravenous immunoglobulin (IVIG) treatment rather than direct coronary artery outcomes. Because IVIG resistance has been associated with an increased risk of coronary complications, these scoring systems have also been explored indirectly for the identification of patients at higher risk for coronary artery involvement. However, studies performed in non-Asian populations have reported inconsistent and often limited performance of these scoring systems, raising concerns regarding their generalizability across different ethnic and geographic settings (2–8). More recently, Türkuçar et al. identified male sex, age ≤12 months, and fever duration before IVIG treatment ≥9.5 days as independent predictors of coronary artery lesions in a large multicenter cohort of Turkish children with KD and proposed a simple population-specific clinical prediction model. However, independent external validation of this model and further evaluation of coronary risk prediction tools in Turkish children remain limited (9).

Data regarding the performance of these risk scoring systems in Turkish children with KD remain limited. In addition, readily available clinical factors associated with coronary artery involvement in this population have not been clearly defined. Therefore, this study aimed to evaluate clinical factors associated with CAA development and to explore the performance of commonly used KD risk scoring systems in a Turkish pediatric cohort.

## 2 Materials and methods

### 2.1 Study design and population

This retrospective single-center cohort study included children diagnosed with Kawasaki disease between January 1994 and December 2019 at a tertiary pediatric referral center in Turkey. Patients were identified through hospital medical records using diagnostic codes and clinical documentation. Kawasaki disease was diagnosed according to the criteria of the American Heart Association. Patients with incomplete clinical records or without available echocardiographic evaluation were excluded from the analysis.

### 2.2 Data collection and definitions

Demographic characteristics, clinical findings, laboratory parameters at diagnosis, treatment details, and follow-up data were extracted from medical records. Fever duration was defined as the total number of days with documented fever prior to IVIG administration. Delayed IVIG treatment was defined as initiation of IVIG on or after the 10th day of illness. Laboratory parameters included white blood cell count, hemoglobin level, platelet count, erythrocyte sedimentation rate, C-reactive protein, alanine aminotransferase, and serum albumin levels obtained at presentation.

Diagnosis was reviewed according to the criteria of the American Heart Association (10). Patients with fever lasting five or more days and at least 4 of the 5 clinical features (1 Erythema and cracks of the lips, strawberry tongue or erythema of the oral pharyngeal mucosa, 2 Bilateral bulbar conjunctivitis without exudates, 3 Maculopapular rash, diffuse erythroderma or erythema multiforme-like rash, 4 Erythema and edema of the extremities in the acute phase, periungual desquamation in the subacute phase, 5 cervical lymphadenopathy ≥1.5 cm in diameter, usually unilateral) were defined as complete Kawasaki Disease. Incomplete disease was diagnosed by examining the patients with fever lasting for five days or more and additional 2-3 criteria, or infants with fever lasting 7 days or more and for whom fever could not be explained by any other reason, according to the following criteria: C reactive protein (CRP) ≥3 mg/dL or erythrocyte sedimentation rate (ESR) ≥40 mm/s, plus 3 laboratory (anemia, platelet count ≥450000/mm^3^, albumin ≤3 g/dL, elevated ALT, leukocyte count ≥15000/mm3, leukocytes in urine ≥10/hpf) or echocardiographic findings, is defined as incomplete Kawasaki Disease.

Resistance to IVIG treatment was defined as persistent fever 48 h after administration of the first dose. Coronary artery measurements were analyzed using the coronary artery z-score method, in accordance with the American Heart Association scientific statement (10). Z score of 2.5 and above was defined as coronary artery aneurysm.

Echocardiographic examinations were performed by pediatric cardiologists using standard techniques. Coronary artery dimensions were normalized for body surface area and expressed as z-scores. Coronary artery aneurysm was defined as a coronary artery z-score ≥2.5 in any major coronary segment during the course of follow-up.

Echocardiography was routinely performed at the time of diagnosis as part of the initial evaluation, with baseline coronary artery assessment obtained before or around the time of IVIG administration whenever feasible. Follow-up echocardiographic examinations were subsequently performed according to institutional practice and contemporary American Heart Association recommendations, and coronary outcomes were determined using the highest coronary artery z-score recorded during follow-up.

### 2.3 Risk scoring systems

Established clinical risk scoring systems commonly used in Kawasaki disease were applied retrospectively using data obtained at diagnosis. Kobayashi, Egami, Sano, Formosa and Harada scoring systems are the systems examined (11–15). These scoring systems included combinations of demographic, clinical, and laboratory variables. Scores were calculated according to their original definitions, and predefined cut-off values were used to categorize patients as high or low risk. Scoring systems are summerized in Table 1. The performance of each scoring system was evaluated for its ability to identify both IVIG resistance and coronary artery aneurysm.

**Table 1:** Kawasaki disease risk scoring systems.

Scoring system	Parameters	Score values	High risk definitions
Kobayashi	Na ≤133 mmol/L	2	≥4 points
	Treatment day ≤4 days	2	
	AST ≥100 IU/L	2	
	Neutrophil ratio ≥%80	2	
	CRP ≥10 mg/dL	1	
	Age ≤12 months	1	
	Plt ≤300000/mm^3^	1	
Egami	Age ≤6 months	1	≥3 points
	Treatment day ≤4 days	1	
	Plt ≤300000/mm^3^	1	
	CRP ≥8 mg/dL	1	
	ALT ≥80 IU/L	2	
Sano	CRP ≥7 mg/dL	1	≥2 points
	TB ≥0.9 mg/dL	1	
	AST ≥200 IU/L	1	
Formosa	Lymphadenopathy	1	≥3 points
	Neutrophil % ≥60	2	
	Albumin <3.5 g/dL	1	
Harada	WBC >12000/mm^3^	1	≥4 points
	Platelets <350000/mm^3^	1	
	CRP >3 mg/dL	1	
	Hematocrit <35%	1	
	Albumin <3.5 g/dL	1	
	Age ≤12 months	1	
	Sex = Male	1	

ALT, alanine aminotransaminase; AST, aspartate transaminase; CRP, C-reactive protein; Plt, platelet count; TB, total bilirubin; WBC, white blood cell count.

Although several of these scoring systems were originally developed to predict IVIG resistance rather than coronary outcomes, all were evaluated for coronary artery aneurysm in this study due to the well-established association between IVIG resistance and coronary artery involvement. Given the objective of identifying patients at increased risk for coronary complications, these scoring systems were applied uniformly for the assessment of coronary artery aneurysm development.

### 2.4 Statistical analysis

Continuous variables were assessed for normality using the Shapiro–Wilk test. Data are presented as mean ± standard deviation or median (interquartile range), as appropriate. Categorical variables are presented as numbers and percentages. Comparisons between patients with and without coronary artery aneurysm were performed using Student's t-test or Mann–Whitney U test for continuous variables and chi-square or Fisher's exact test for categorical variables, as appropriate. Receiver operating characteristic (ROC) curve analysis was performed to assess the discriminatory ability of fever duration for predicting coronary artery aneurysm. A two-sided *p* value <0.05 was considered statistically significant. Statistical analyses were performed using SPSS software version 22 (version 22; IBM Corp., Armonk, NY, USA).

### 2.5 Ethical considerations

The study was approved by Ankara University Faculty of Medicine Human Research Ethics Committee on 13.05.2019 (approval number: 09-692-19). Due to the retrospective nature of the study, the requirement for informed consent was waived.

## 3 Results

### 3.1 Patient characteristics

A total of 81 children diagnosed with Kawasaki disease were included in the final analysis. Among the initially screened patients, 7 were excluded because of incomplete clinical records or unavailable echocardiographic data. The median age at diagnosis was 2.61 years (IQR: 1.19–4.31 years), and 30 patients (37%) were female. The male-to-female ratio was 1.7:1.

Complete Kawasaki disease was observed in 56 patients (69.1%), whereas 25 patients (30.9%) were classified as having incomplete Kawasaki disease.

All patients received standard initial treatment with high-dose IVIG. IVIG resistance was observed in 13 patients (16%). Patients with IVIG resistance received a second dose of IVIG, and clinical response was achieved in all cases.

### 3.2 Coronary outcomes

Coronary artery aneurysm developed in 18 of 81 patients (22.2%) during the acute or follow-up period. Coronary outcomes were determined based on the highest recorded coronary artery z-score identified during serial echocardiographic evaluations. CAA was defined as a coronary artery z-score ≥2.5 in at least one major coronary artery segment.

Among patients with CAA, regression of coronary abnormalities during follow-up was observed in 13 patients (72.2%). Persistent coronary artery abnormalities at the last available echocardiographic evaluation were present in the remaining patients.

### 3.3 Risk factors for coronary artery aneurysm

Clinical and laboratory characteristics of patients with and without coronary artery aneurysm are summarized in Table 2. Younger age at diagnosis, prolonged fever duration, and delayed initiation of IVIG treatment were more frequently observed in patients who developed coronary artery aneurysm. No statistically significant associations were observed regarding sex, disease presentation (complete vs. incomplete Kawasaki disease), or IVIG resistance.

**Table 2:** Clinical features of patients with/without coronary artery aneurysm.

Variable		Patient without CAA	Patient with CAA	p
Age	(Median)	2.61	2.67	>0.05
Gender	Female *n* (%)	20 (66.7)	10 (33.3)	>0.05 >0.05
	Male *n* (%)	43 (84.3)	8 (15.6)	
Complete/Incomplete	Complete *n* (%)	46 (82.1)	10 (17.9)	>0.05
	Incomplete *n* (%)	17 (68)	8 (32)	>0.05
Laboratory Data	Hb (g/dL)	10.2	9.6	>0.05
	WBC (/mm^3^)	15800	18500	>0.05
	Plt (/mm^3^)	632000	710000	>0.05
	CRP (mg/L)	132.2	153.8	>0.05
	ESR (mm/s)	79.2	99.1	0.045
Other	Valvular involvement	11.1%,	35%	0.034

CRP, C-reactive protein; Hb, hemoglobin; Plt, platelet count; TB, total bilirubin; WBC, white blood cell count.

No consistent association was observed between routine laboratory parameters and coronary artery aneurysm development.

ROC curve analysis demonstrated moderate discriminatory ability of fever duration for prediction of coronary artery aneurysm (AUC: 0.74). A fever duration cut-off value of 9 days provided the optimal balance between sensitivity and specificity.

Patients aged 6 months or younger showed a higher frequency of coronary artery aneurysm compared with older children. Patients aged 6 months or younger, prolonged fever duration, and delayed IVIG treatment were more frequently observed among patients who developed coronary artery aneurysm. (Table 3).

**Table 3:** Association of clinical factors with coronary artery aneurysm.

Variable	Coronary artery aneurysm	P
Delayed IVIG treatment (IVIG >10th illness day)	80%	0.005
IVIG treatment day ≤10 days	15.9%	
Total fever duration >10 days	63.6%	0.001
Total fever duration ≤10 days	14.5%	
Age ≤6 months	100%	0.001
Age >6 months	19.2%	

### 3.4 Performance of risk scoring systems for predicting coronary artery aneurysm

Sensitivity for coronary artery aneurysm ranged from 13% to 92%, whereas specificity ranged from 15% to 76% across the evaluated scoring systems. Some scoring systems demonstrated relatively higher sensitivity with limited specificity, whereas others showed higher specificity at the expense of sensitivity. Overall, no scoring system demonstrated balanced diagnostic performance for the prediction of coronary artery aneurysm in this cohort. Detailed performance characteristics are summarized in Table 4. Because these scoring systems were originally developed primarily for prediction of IVIG resistance rather than direct coronary artery outcomes, their performance for coronary artery aneurysm prediction should be interpreted cautiously.

**Table 4:** Characteristics of scoring systems associated with the development of coronary artery aneurysm.

Scoring system	Sensitivity %	Specificity %	Positive likelihood ratio	Negative likelihood ratio	Accuracy %
Kobayashi	26.7	69.5	0.87	1.06	60.74
Egami	13.3	76.3	0.56	1.14	63.51
Sano	21.4	76.8	0.92	1.02	65.71
Formosa	64.3	35.7	0.98	1.04	40.28
Harada	92.9	15.5	1.1	0.46	30.56

We additionally evaluated the three clinical predictors proposed by Türkuçar et al. (male sex, age ≤12 months, and fever duration before IVIG ≥9.5 days) in our cohort. Age ≤12 months and fever duration before IVIG ≥9.5 days were associated with increased odds of coronary artery aneurysm development, whereas male sex was not associated with coronary involvement. Owing to the relatively small sample size, none of these associations reached statistical significance (Table 5).

**Table 5:** External evaluation of the clinical predictors proposed by Türkuçar et al.

Variable	OR	95% CI	*p* value
Male sex	0.91	0.26–2.65	0.752
Age ≤12 months	2.64	0.77–10.06	0.120
Fever duration before IVIG ≥ 9.5 days	2.47	0.67–10.85	0.161

### 3.5 Performance of risk scoring systems for predicting IVIG resistance

Among the 81 patients included in the study, 13 (16.0%) were classified as IVIG-resistant. The performance of the Kobayashi, Egami, Sano, Formosa, and Harada scoring systems for predicting IVIG resistance is summarized in Table 6. Overall, the scoring systems demonstrated variable sensitivity and specificity, with generally lower predictive performance than originally reported in East Asian populations.

**Table 6:** Performance of kawasaki disease risk scoring systems for prediction of IVIG resistance.

Scoring system	Sensitivity %	Specificity %	Positive likelihood ratio	Negative likelihood ratio	Accuracy %
Kobayashi	38.5	86.7	2.89	0.71	79.06
Egami	53.8	85.0	3.59	0.54	79.94
Sano	53.8	83.9	3.34	0.55	79.03
Formosa	76.9	36.2	1.21	0.64	42.74
Harada	84.6	13.8	0.98	1.12	25.19

## 4 Discussion

In this retrospective single-center cohort of children with Kawasaki disease, coronary artery aneurysm developed in a considerable proportion of patients. Younger age, prolonged fever duration, and delayed initiation of intravenous immunoglobulin treatment were associated with coronary artery aneurysm development. In addition, commonly used Kawasaki disease risk scoring systems demonstrated limited and unbalanced performance for prediction of coronary artery aneurysm in this cohort.

The frequency of coronary artery aneurysm in our cohort (22.2%) was higher than that reported in many contemporary international cohorts, in which CAA rates generally range from approximately 3% to 17%, but lower than those reported in several previous Turkish single-center series, where rates between 26.5% and 38.6% have been described (16–18). These differences should be interpreted in the context of variations in study populations, referral patterns, diagnostic practices, timing of IVIG treatment, and the long study period of our cohort (1994–2019), which spans different eras of Kawasaki disease management. Overall, the coronary outcomes observed in our study are consistent with previously reported data from Türkiye and support the representativeness of our cohort within the national setting.

Previous studies have reported higher rates of coronary artery complications among patients with incomplete Kawasaki disease and IVIG resistance (10). In our cohort, however, no statistically significant association was observed between incomplete disease presentation or IVIG resistance and coronary artery aneurysm development. These differences may be related to variations in study populations, treatment practices, and the relatively limited sample size of the present study.

Male sex has been suggested as a risk factor for worse prognosis and coronary involvement in Kawasaki disease (19, 20). Although Kawasaki disease was more common among male patients in our cohort, no significant difference in coronary artery aneurysm development was observed between sexes. Younger age has consistently been identified as an important prognostic factor in previous studies, particularly in infants younger than 6 or 12 months (11, 12, 19, 20). While overall age was not significantly associated with coronary artery aneurysm development in our study, patients aged 6 months or younger showed a significantly higher rate of coronary involvement, supporting earlier observations that very young infants represent a high-risk subgroup.

Several inflammatory laboratory markers have previously been associated with coronary artery involvement in Kawasaki disease (19–20). In the present cohort, no consistent association was observed between routine laboratory parameters and coronary artery aneurysm development. These findings should be interpreted cautiously because of the retrospective design and limited sample size.

Timely administration of IVIG remains a cornerstone of Kawasaki disease management. In our cohort, prolonged fever duration and delayed IVIG treatment were associated with coronary artery aneurysm development, supporting previous observations emphasizing the importance of early recognition and treatment in Kawasaki disease.

The Kobayashi, Egami, Sano, Formosa, and Harada scoring systems demonstrated variable sensitivity and specificity for prediction of coronary artery aneurysm in our cohort. Some scoring systems showed relatively higher sensitivity with limited specificity, whereas others demonstrated higher specificity at the expense of sensitivity. Overall, no scoring system demonstrated balanced diagnostic performance. An important consideration when interpreting these findings is that most of the evaluated scoring systems were originally developed for prediction of IVIG resistance rather than direct coronary artery outcomes. Although IVIG resistance and coronary artery involvement are clinically associated, these outcomes are not interchangeable. In the present cohort, the evaluated scoring systems also demonstrated only modest performance for predicting IVIG resistance, despite this being their original intended outcome. These findings are consistent with previous studies from non-East Asian populations reporting reduced performance of Japanese-derived risk scores outside their derivation cohorts. Therefore, the limited ability of these scoring systems to predict coronary artery aneurysm in our study is unlikely to be explained solely by differences between IVIG resistance and coronary outcomes, but may also reflect population-specific clinical and genetic characteristics. Therefore, the limited performance observed in our cohort may partly reflect conceptual differences between the original intended purpose of these scoring systems and the outcome evaluated in this study. Differences in ethnicity, genetic background, disease phenotype, and healthcare practices may also contribute to variations in scoring system performance across populations (19, 20).

Our findings should also be interpreted in the context of the recent multicenter Turkish study by Türkuçar et al., which identified male sex, age ≤12 months, and fever duration before IVIG treatment ≥9.5 days as independent predictors of coronary artery lesions and proposed a simple population-specific clinical prediction model (9). Similar to their findings, prolonged fever duration and delayed IVIG treatment were associated with coronary artery aneurysm development in our cohort. In addition, both studies highlighted younger age as an important clinical predictor, although our analysis identified a significant association particularly among infants aged ≤6 months. Unlike Türkuçar et al., male sex was not significantly associated with coronary artery aneurysm in our cohort, which may reflect the smaller sample size and single-center design of our study. Taken together, these findings support the need for population-specific approaches when assessing coronary artery risk in children with Kawasaki disease.

A small number of patients demonstrated persistent coronary artery abnormalities during follow-up. Because of the limited number of cases, further analysis regarding predictors of persistent coronary involvement was not feasible.

The strengths of this study include the evaluation of multiple commonly used Kawasaki disease risk scoring systems within the same cohort and the use of standardized z-score-based coronary artery assessment. In addition, the study provides data from a Turkish pediatric population, contributing to the limited literature regarding the performance of these scoring systems outside East Asian populations.

This study has several limitations. First, its retrospective single-center design limits generalizability and may introduce selection bias. Patients with incomplete clinical records or unavailable echocardiographic data were excluded from analysis, which may have affected cohort composition. Second, the relatively limited sample size, particularly the number of patients with coronary artery aneurysm, restricted the statistical power of subgroup analyses. Third, the prolonged study period spanning 1994–2019 may have introduced heterogeneity related to temporal changes in diagnostic criteria, echocardiographic imaging quality, coronary artery z-score assessment, and treatment strategies. Finally, the evaluated scoring systems were originally developed primarily for prediction of IVIG resistance rather than direct coronary artery outcomes, which should be considered when interpreting their performance in this cohort. In addition, although the Son score has recently been recommended by the 2024 American Heart Association Scientific Statement for coronary artery aneurysm risk stratification, it could not be evaluated because baseline coronary artery z-scores were not systematically available for all patients in this retrospective cohort. Analysis of only patients with available z-scores would have introduced substantial selection bias. Finally, although echocardiographic evaluation was routinely performed at diagnosis and repeated during follow-up, the retrospective design and long study period may have resulted in variation in the timing of imaging, potentially leading to underestimation of transient coronary artery dilatation in a small number of patients.

In conclusion, commonly used clinical risk scoring systems demonstrated limited performance for predicting coronary artery aneurysm in this Turkish cohort of children with Kawasaki disease. Very young age, prolonged fever duration, and delayed IVIG treatment were associated with coronary involvement and may provide useful clinical cues for risk assessment. Larger, prospective, multicenter studies are needed to develop or validate population-specific tools for predicting coronary complications in Kawasaki disease.

## Data Availability

The raw data supporting the conclusions of this article will be made available by the authors, without undue reservation.
